# Inhibition of Inactive States of Tetrodotoxin-Sensitive Sodium Channels Reduces Spontaneous Firing of C-Fiber Nociceptors and Produces Analgesia in Formalin and Complete Freund’s Adjuvant Models of Pain

**DOI:** 10.1371/journal.pone.0138140

**Published:** 2015-09-17

**Authors:** David J. Matson, Darryl T. Hamamoto, Howard Bregman, Melanie Cooke, Erin F. DiMauro, Liyue Huang, Danielle Johnson, Xingwen Li, Jeff McDermott, Carrie Morgan, Ben Wilenkin, Annika B. Malmberg, Stefan I. McDonough, Donald A. Simone

**Affiliations:** 1 Department of Neuroscience, Amgen Inc., Cambridge, Massachusetts, United States of America; 2 Department of Medicinal Chemistry, Amgen Inc., Cambridge, Massachusetts, United States of America; 3 Department of Pharmaceutics Research & Development, Amgen Inc., Cambridge, Massachusetts, United States of America; 4 Department of Pharmacokinetics & Drug Metabolism, Amgen Inc., Cambridge, Massachusetts, United States of America; 5 Department of Diagnostics and Biological Sciences, University of Minnesota School of Dentistry, Minneapolis, Minnesota, United States of America; Xuzhou Medical College, CHINA

## Abstract

While genetic evidence shows that the Nav1.7 voltage-gated sodium ion channel is a key regulator of pain, it is unclear exactly how Nav1.7 governs neuronal firing and what biophysical, physiological, and distribution properties of a pharmacological Nav1.7 inhibitor are required to produce analgesia. Here we characterize a series of aminotriazine inhibitors of Nav1.7 *in vitro* and in rodent models of pain and test the effects of the previously reported “compound 52” aminotriazine inhibitor on the spiking properties of nociceptors *in vivo*. Multiple aminotriazines, including some with low terminal brain to plasma concentration ratios, showed analgesic efficacy in the formalin model of pain. Effective concentrations were consistent with the *in vitro* potency as measured on partially-inactivated Nav1.7 but were far below concentrations required to inhibit non-inactivated Nav1.7. Compound 52 also reversed thermal hyperalgesia in the complete Freund’s adjuvant (CFA) model of pain. To study neuronal mechanisms, electrophysiological recordings were made *in vivo* from single nociceptive fibers from the rat tibial nerve one day after CFA injection. Compound 52 reduced the spontaneous firing of C-fiber nociceptors from approximately 0.7 Hz to 0.2 Hz and decreased the number of action potentials evoked by suprathreshold tactile and heat stimuli. It did not, however, appreciably alter the C-fiber thresholds for response to tactile or thermal stimuli. Surprisingly, compound 52 did not affect spontaneous activity or evoked responses of Aδ-fiber nociceptors. Results suggest that inhibition of inactivated states of TTX-S channels, mostly likely Nav1.7, in the peripheral nervous system produces analgesia by regulating the spontaneous discharge of C-fiber nociceptors.

## Introduction

Voltage-gated sodium channels are major drivers of the excitability of sensory neurons, including those encoding noxious inputs. Many of the nine individual sodium channel subtypes expressed in the peripheral nervous system have distinct biophysical properties [1], and it is not known what roles these different subtypes might play in the response properties of different neuronal types *in vivo* [2]. This is particularly true for persistent and chronic pain conditions, in which neurons are hyper-excitable and may encode normally non-noxious stimuli as noxious due to altered gene expression, neuronal connectivity, or second-messenger signaling [3, 4].

Sodium channel pharmacology is advancing rapidly, driven by the intense interest in sodium channels as potential targets for new therapeutics to treat pain [5–7]. Most current interest focuses on developing subtype-selective inhibitors of Nav1.7 and arises from genetic data showing that loss of Nav1.7 function significantly attenuates pain, with anosmia the only apparent additional phenotype [8–14]. Accordingly, subtype-specific Nav1.7 blockers are being explored by many companies, but the path is still being developed as to which gating states of the channel to inhibit, which *in vitro* parameters drive *in vivo* efficacy, which *in vivo* driver models are most appropriate, and whether CNS penetration is required to produce analgesic efficacy [15, 16]. Current efforts have produced a variety of new compounds with a degree of *in vitro* selectivity, some of which are suitable for use as *in vivo* tools [17]. For a given compound, parallel comparison of 1) *in vitro* selectivity and biophysical properties; 2) effects in models of pain; and 3) effects on the firing of nociceptive neurons can contribute to the understanding of mechanistic roles of different sodium channels and different neuronal fiber types in pain.

In this study, pain behaviors and the activity of nociceptive neurons *in vivo* were examined in response to systemic administration of “compound 52,” an aminotriazine Nav1.7 inhibitor with some CNS penetration appropriate for *in vivo* use. While tools for true Nav1.7-selective inhibition *in vivo* are not yet available, compound 52 has approximate tenfold selectivity for TTX-S sodium channels against TTX-R sodium channels. It is strongly state dependent, more strongly than the local anesthetic mexiletine, with extrapolated 100-fold preference for inactivated versus non-inactivated Nav1.7 [18]. Effects of compound 52 and of close congeners with varying degrees of CNS penetration were evaluated in the formalin model of pain, a high-throughput model reflecting noxious chemical input. Compound 52 was further tested in the CFA model of persistent inflammation-induced pain and a nerve injury model, and was also tested for effects on the response properties of single nociceptors from the tibial nerve of rats treated with CFA. Results show a specificity to the effects of compound 52 on Nav1.7 gating states and on firing patterns of different neuronal fiber types. Results suggest that inactivated states of TTX-S Navs (most likely Nav1.7) govern pain by regulating spontaneous and evoked spiking, with minimal effect on response thresholds, of C-fiber nociceptors, but have little effect on Aδ-fiber nociceptors.

## Materials and Methods

Adult male Sprague-Dawley rats (Harlan, Frederick, MD) weighing 270–360 g at the time of testing were used. All animals were housed in groups of two on solid bottom Plexiglas cages (38 x 28 x 19 cm) with free access to food and water in a temperature- and humidity- controlled room on a 12 / 12 hour light / dark cycle. All protocols were approved by Amgen Inc.’s Institutional Animal Care and Use Committee, or by the Institutional Animal Care and Use Committee at the University of Minnesota.

### Behavioral studies

#### Drugs

Morphine sulfate (Sigma, St. Louis, MO) was dissolved in Phosphate Buffered Saline (PBS) and dosed subcutaneously (s.c.) 30 minutes before testing. Aminotriazine compounds were synthesized by Amgen, Inc. (Cambridge MA). For oral administration (p.o.) delivery, compounds were dissolved in 30% hydroxypropyl-β-cyclodextrin (HP-B-CD) pH-adjusted with methanesulfonic acid and dosed 120 minutes before testing. Formulation excipient HP-B-CD was purchased from Ashland. Methanesulfonic acid was purchased from Fluka or Sigma. Except as indicated, all behavioral studies were done with p.o. delivery. For intravenous (i.v.) delivery of compound 52, the drug substance was formulated in 30% HP-B-CD in water pH 3 as a solution at 5 mg/mL and delivered in a total bolus of 500 microliters, for a total dose of 2.5 mg (approximately 8 mg/kg depending on the weight of the animal). Compound 52 or vehicle control was delivered via the external jugular vein in electrophysiological studies.

**Fig 1:**
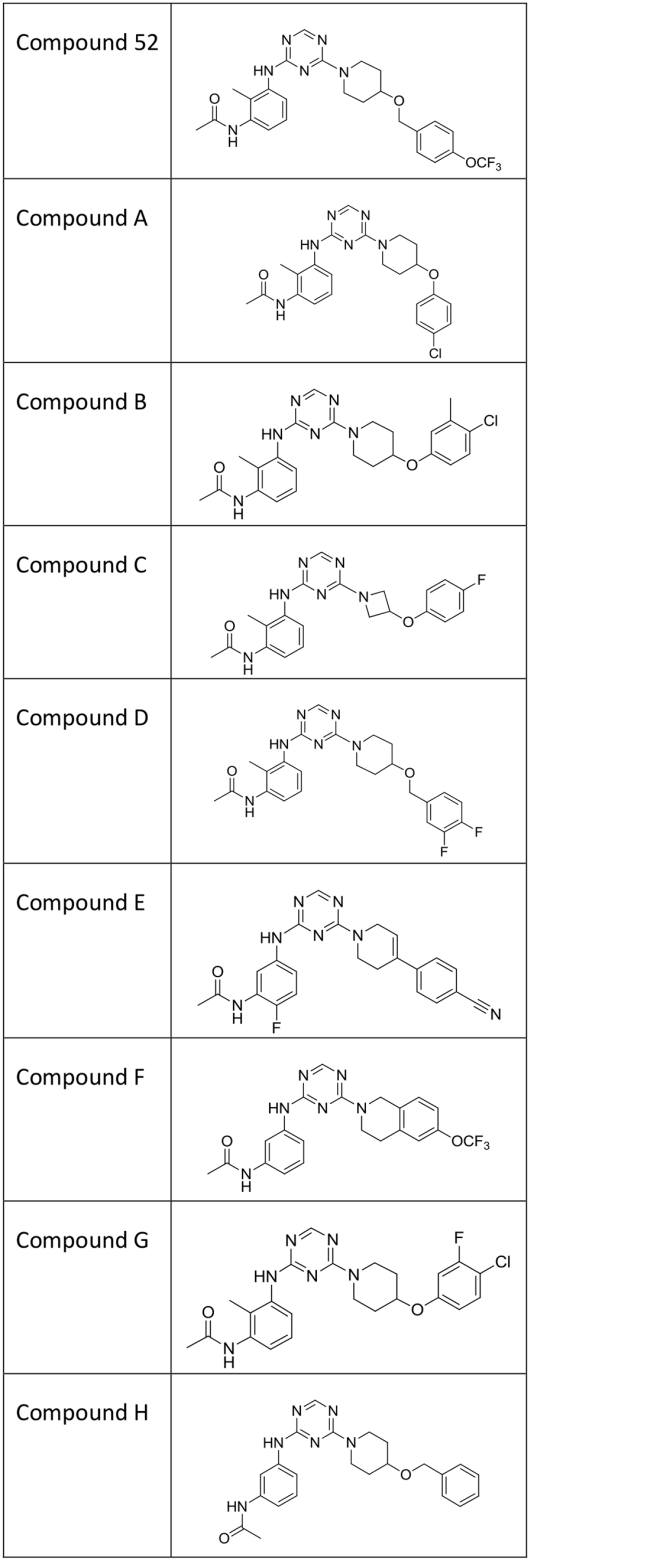
Structures of aminotriazine compounds used in this study.

Aminotriazines are named in the text as compound 52 and as compounds A through H, with structures shown in Fig 1. IUPAC compound names are as follows. Compound 52 is N-(2-methyl-3-((4-(4-((4-(trifluoromethoxy)benzyl)oxy)-1-piperidinyl)-1,3,5-triazin-2-yl)amino)phenyl)acetamide. Compound A is N-(3-((4-(4-(4-chlorophenoxy)-1-piperidinyl)-1,3,5-triazin-2-yl)amino)-2-methylphenyl)acetamide. Compound B is N-(3-((4-(4-(4-chloro-3-methylphenoxy)-1-piperidinyl)-1,3,5-triazin-2-yl)amino)-2-methylphenyl)acetamide. Compound C is N-(3-((4-(3-(4-fluorophenoxy)-1-azetidinyl)-1,3,5-triazin-2-yl)amino)-2-methylphenyl)acetamide. Compound D is N-(3-((4-(4-((3,4-difluorobenzyl)oxy)-1-piperidinyl)-1,3,5-triazin-2-yl)amino)-2-methylphenyl)acetamide. Compound E is N-(5-((4-(4-(4-cyanophenyl)-3,6-dihydro-1(2H)-pyridinyl)-1,3,5-triazin-2-yl)amino)-2-fluorophenyl)acetamide. Compound F is N-(3-((4-(6-(trifluoromethoxy)-3,4-dihydro-2(1H)-isoquinolinyl)-1,3,5-triazin-2-yl)amino)phenyl)acetamide. Compound G is N-(3-((4-(4-(4-chloro-3-fluorophenoxy)-1-piperidinyl)-1,3,5-triazin-2-yl)amino)-2-methylphenyl)acetamide. Compound H is N-(3-((4-(4-(benzyloxy)-1-piperidinyl)-1,3,5-triazin-2-yl)amino)phenyl)acetamide. Compound H was previously referred to as “compound 36” in our initial report of aminotriazines [18]. Although compounds A through G were not described in that paper, they were prepared with the general protocols reported therein.

#### Formalin test

Formalin-induced flinching was measured using the Automated Nociception Analyzer (ANA) (University Anesthesia Research & Development Group, La Jolla, CA). Rats were habituated to the ANA testing chambers for at least 30 minutes prior to the formalin injection. At test time, each rat was removed from the testing chamber and gently wrapped in a towel. 50 μL of 2.5% formalin solution was injected into the dorsal surface of the left hind paw with a 30 gauge needle. A small metal band was then affixed to the plantar side of the injected paw with one drop of super glue. The animal was then unwrapped from the towel, placed back in the testing chamber, and flinching behavior was scored for 40 minutes by the ANA software. While minimized, non-painful paw movement from the natural activity of an alert animal could be erroneously binned by the automated recording system as a nociceptive behavior. These behaviors are not subtracted from the flinching total. Hence the total flinches for a naïve animal would not necessarily be zero. Total flinching behavior was used as the endpoint of the assay.

#### Open field activity

Basic movement was measured using the Photobeam Activity System Open Field (San Diego Instruments, San Diego, CA) equipped with 48 infrared photocell emitters and detectors. Photobeams used to measure vertical movement were positioned 14 cm above the test chamber floor. Each testing chamber was made of Plexiglas (41 x 41 x 36 cm) and was devoid of any bedding. At the beginning of the test session, each animal was placed in a separate testing chamber and the room lights were turned off. Basic movement was measured for 30 minutes.

#### Thermal hyperalgesia following inflammation

CFA-induced thermal hyperalgesia was measured using the Thermal Plantar Analgesia Instrument (Ugo Basile, Italy). Intensity of the radiant heat stimulus was adjusted to achieve a withdrawal latency of approximately 13 s in naïve animals. On test day one, animals were placed on the apparatus in their individual testing chambers with an absorbent towel at the bottom and allowed to habituate for at least 30 minutes. The towel was then removed and animals were allowed to acclimate for at least 15 minutes more. After habituation, animals were placed back in their home cages until the following day. On test day two, animals were placed back in the testing chambers until calm (typically 20 minutes). The thermal device was positioned on the center of the left hind paw and the test was started. The time for the animal to withdraw its paw from the thermal stimulus was recorded. A cut-off of 30 seconds was used to prevent tissue damage. Few trials went past 20 seconds and none past 25 seconds. This procedure was repeated for three trials per animal, each separated by at least 5 min, and trials were averaged to determine baseline withdrawal latencies. After determining baseline measures, each animal received an injection of 50 μl CFA (1:1 emulsion with PBS, Sigma, St. Louis, MO) into the plantar side of the left hind paw with a 26 gauge needle. Animals were then placed back in their home cages until the following day. Approximately 24 h after the CFA injection, withdrawal latencies to heat were determined as described above. Animals were then randomized based on their Post-CFA baseline score and treated with the test compound or vehicle. Withdrawal latency averages were again determined at 120 minutes (compound 52) or at 30 minutes (morphine) following compound administration. The experimenter was blinded to drug treatment during this assay. Percent return to baseline latency was used as the endpoint of the assay.

#### Tactile allodynia following nerve injury

A decrease in tactile response threshold, using calibrated von Frey filaments (Stoelting Co., Wood Dale, IL), was used as a measure of tactile allodynia following spinal nerve ligation (SNL). Prior to surgery, each animal underwent baseline testing to determine suitability for the assay. Briefly, animals were placed on a raised platform with a grid meshed floor. The distance between the mesh was ¼”. Once acclimated, each animal received a series of trials according to the up-down method described by Dixon [19]. Animals showing a response to less than 4 g were deemed already sensitive to the stimulus and were removed. A maximal fiber of 15 g was employed as a cut-off threshold. Surgery was then performed with a procedure slightly modified by that reported by Kim and Chung [20]. Briefly, the left L5 spinal nerve was carefully isolated from the surrounding tissue. Using 5–0 silk suture, a tight ligature was placed around L5 nerve. Muscle and skin were then closed using 4–0 absorbable suture and wound clips. Animals were allowed to recover and were carefully monitored for seven days to ensure proper healing and ensure there was no mutilation of the affected limb. On day 21 post-surgery, animals were again tested to ensure the development of tactile allodynia. Animals that did not exhibit at least a 5 g decrease in response threshold were not included in the study. Animals were then randomized into groups and dosed with the test compound, gabapentin, or vehicle and assessed as stated above. Paw withdrawal thresholds were determined before and either 75 minutes (gabapentin) or 120 minutes (compound 52 and vehicle) after dosing. The experimenter was blinded to drug treatment during this assay. Percent return to baseline was used as the endpoint of this assay.

### Patch clamp electrophysiology

Except as indicated, recordings to determine potency of aminotriazines on Nav1.7 were made with manual electrophysiology. HEK 293 cells stably expressing hNav1.7 were voltage-clamped using the whole cell patch clamp configuration. Cells were plated onto 35 mm culture dishes 0–24 hours before recording, and tissue culture medium was exchanged for external solution containing (in mM) 140 NaCl, 5 KCl, 2 CaCl2, 1 MgCl2, 10 HEPES, 11 glucose, pH 7.4 with NaOH. Patch pipettes were pulled from borosilicate glass capillaries (1.2 mm outer diameter, World Precision Instruments, Sarasota, FL) with a P-97 pipette puller (Sutter Instruments, Novato, CA) and backfilled with internal solution containing (in mM) 62.5 CsCl, 75 CsF, 2.5 MgCl2, 5 EGTA, 10 HEPES, pH 7.25 with CsOH. Pipette resistances were between 1.5 and 2.0 MΩ. After formation of the whole-cell configuration, cells were lifted off the dish bottom with the patch pipette and positioned directly in front of a microarray of glass tubes (each tube internal diameter ~ 1 mm) with each tube containing continuously flowing control or test solution. A full current-voltage relation was recorded from each cell, and cells with poor space clamp as indicated by discontinuous I-V relation or by prolonged or nonexponential current kinetics were discarded. Solution switching was accomplished by moving the microarray with computer control via an RSC-160 rapid solution exchanger (Bio-Logic, Claix, France). Currents were recorded with an Axopatch 200B patch-clamp amplifier driven by pCLAMP software, filtered (4-pole Bessel) at 5 kHz during acquisition, and digitized at 20 kHz using pClamp9.2. Whole cell capacitance and series resistance were compensated at 0%- 80%, and reported voltages were uncorrected for liquid junction potentials. Recordings were made at room temperature. Concentration-response curves were taken by measuring inward Nav1.7 current every five to six seconds with a 10 ms to 20 ms depolarization to near the peak of the current-voltage relation, before and after steady-state inhibition by a given concentration of aminotriazine. For concentration response curves on partially inactivated channels, holding voltage was set to produce 20% fractional inactivation. After each concentration had reached steady-state inhibition, compound was washed off (usually with holding voltage switched to -120 mV or -140 mV), and the holding voltage was then reset as needed to a value that produced 20% fractional inactivation for testing of the next compound concentration. Occasionally two compound concentrations were tested in parallel without resetting the voltage, if inhibition was rapid and the current level steady. Concentration response curves for non-inactivated channels were measured with a holding voltage of -140 mV or occasionally -120 mV [21], and in all cases voltages were checked to ensure that a holding voltage 10 mV more positive did not produce inactivation. Concentration response curves on non-inactivated channels generally were taken with multiple concentrations added in series without washoff of the previous concentration. Peak concentration of compound tested was ten micromolar, since at higher concentrations aminotriazines could fall out of solution. For non-inactivated channels and 20% inactivated channels, four-point IC50 values were fit to concentration-response curves using the Hill equation and force-fitting a slope of unity with at least n = 2 cells per concentration. IC50 values for rat Nav1.7 channels, with holding voltage set to produce 20% fractional inactivation, were determined with the PatchXpress automated electrophysiology platform as described [18].

### *In vivo* electrophysiological studies

A total of 55 adult, male, Sprague–Dawley rats (Harlan, Indianapolis, IN) weighing 300–350 g were used for electrophysiology. To ensure that electrophysiology studies were performed on rats with mechanical hyperalgesia, behavioral measures of mechanical hyperalgesia were obtained before and at 24 h after administration of CFA (and just prior to the electrophysiological studies). Mechanical hyperalgesia was assessed by determining the frequency of paw withdrawal evoked by mechanical stimulation using a 26 g Semmes-Weinstein von Frey monofilament (Stoelting, Wood Dale, IL). Rats were placed on an elevated wire mesh platform under individual plastic cages and acclimated to the testing environment for 30 minutes prior to testing. The monofilament was applied to the mid-plantar surface of the hindpaw ten times for 1–2 s each with an inter-stimulus interval of approximately 5–10 s and the paw withdrawal frequency (percent) was determined. Baseline measures were determined for each rat for three consecutive days prior to injection of CFA. Mechanical hyperalgesia was defined as an increase in the paw withdrawal frequency.

#### Surgical preparation

Rats were initially anesthetized by intramuscular injection of ketamine (100 mg/kg) and xylazine (45 mg/kg). The trachea was cannulated and a catheter was placed in the external jugular vein to provide supplemental anesthesia with sodium pentobarbital (10 mg/kg/h) and for injection of vehicle or compound 52. Core body temperature was maintained at 37°C using a feedback-controlled heating pad (Harvard Apparatus, Holliston, MA, USA).

#### Electrophysiological recording

Recordings were made from cutaneous afferent fibers of the left tibial nerve using a teased-fiber approach. The tibial nerve was dissected from the surrounding tissue and the overlaying skin was sewn to a metal ring to form a pool that was filled with warm mineral oil. The tibial nerve was placed onto a mirror platform for fine dissection with sharpened fine forceps (Fine Science Tools, Foster City, CA, USA). Teased fibers were placed onto a tungsten wire electrode and action potentials were recorded extracellularly. Action potentials were amplified, audio monitored, displayed on an oscilloscope, and stored on a PC computer for data analysis. Only fibers with clearly discriminated single action potentials were studied. Responses of individual fibers were analyzed off-line using the Spike2 data analysis program (Cambridge Electronic Design, Cambridge, UK).

#### Identification of afferent fibers

Afferent fibers were found by their ongoing spontaneous activity and by mechanically stimulating the plantar surface of the hind paw with the experimenter's finger or von Frey monofilaments. Once a single fiber was identified that was excited by mild pinching but not light touch, its mechanical receptive field was marked on the skin using a felt-tipped pen.

#### Conduction velocity

Conduction velocity was determined by electrically stimulating the skin outside the fiber’s receptive field with pin electrodes (200 μs pulse width at 0.5 Hz) to electrically activate the fiber. The fiber was stimulated at 1.5 times its electrical threshold and the conduction latency was measured from the time of the electrical stimulus artifact to the evoked action potential. Conduction distance was determined by measuring the distance from the fiber's receptive field to the recording electrode. Conduction velocity (m/s) was calculated by dividing conduction distance by conduction latency. Fibers were classified as Aδ-fiber nociceptors if they had conduction velocities between 2.4–25.0 m/s and as C-fiber nociceptors if conduction velocity was < 2.4 m/s [22].

#### Functional classification of nociceptors

Nociceptors were classified functionally according to their responsiveness to mechanical and heat stimulation. Mechanical stimuli used to classify units included light brushing with the tip of a cotton swab, mildly pinching with a pair of forceps, and application of von Frey filaments. Mechanical response thresholds were determined using a series of von Frey monofilaments and defined as the weight (g) required to evoke at least one action potential when applied to the receptive field for 1–2 s. Heat stimuli were delivered using a feedback-controlled Peltier device (Yale Electronics, New Haven, CT) with a contact area of 1 cm^2^. Heat stimuli of 34–50°C, each of 5 s duration, were delivered in ascending order of 2°C with an inter-stimulus interval of 60 s.

#### Drug preparation and administration

Compound 52 was dissolved as described above and administered in a total volume of 500 microliters at a concentration of 5 mg/mL, for a total dose of 2.5 mg. This produced a dose range of 7.1 mg/kg to 8.3 mg/kg for rats of the weight used here. Compound was injected slowly, over a period of 15 min.

#### Experimental design

Once a nociceptor was characterized, ongoing spontaneous activity (Hz) was recorded for a period of 5 min and baseline responses evoked by a 26 g von Frey monofilament and by the series of heat stimuli were determined. The von Frey filament was usually above the mechanical response threshold for nociceptors sampled in this study and was the same filament used to determine mechanical hyperalgesia in our behavioral studies. This monofilament was applied for 5 sec three times to the same location with an inter-stimulus interval of approximately 30 s. The number of evoked impulses was averaged over the three trials. Next, the ascending series of heat stimuli were delivered and heat response threshold was defined as the lowest intensity of consecutive stimulus temperatures to evoke an increase in ongoing activity. The number of impulses evoked by each stimulus temperature was determined. After baseline measures of ongoing spontaneous activity and evoked responses were determined, either vehicle or compound 52 was given intravenously over a period of 15 min. Ongoing spontaneous activity was determined continuously for a 2 min period before injection, during injection, and for 15 min after injection. Responses evoked by mechanical and heat stimuli were determined at 30, 60 and 90 minutes after injection.

#### Data analyses

For *in vivo* studies, data are represented as means +/- SEM of 8–10 animals per group. Overall analyses of the formalin and mobility measurement assays were performed with a one-way ANOVA. Follow-up group comparisons were conducted using Dunnett’s multiple comparison test, with significance set at p = 0.05 or lower. Overall analyses of the CFA-Induced Thermal Hyperalgesia and Spinal Nerve Ligation assays were performed using a two-way ANOVA. Follow-up group comparisons were performed using one-way ANOVAs and the Bonferroni post-hoc test, with significance set at p = 0.05 or lower. Significance compared to vehicle: * represents p < 0.05, ** represents p < 0.01, and *** represents p < 0.001. All analyses were performed on GraphPad Prism, Version 5 (GraphPad Software, La Jolla, CA) or JMP, Version 9.0.0 (SAS Institute, Cary, NC). For electrophysiology studies, ongoing spontaneous activity (Hz) for each nociceptor was divided into 2-min bin widths. Differences in ongoing spontaneous activity before and after injection of vehicle or compound 52 were determined using a two-way ANOVA with repeated measures. Post-hoc comparisons of changes in ongoing spontaneous activity over time and between drug conditions were determined using Newman-Kuels post hoc tests. One-way ANOVAs with repeated measures were also used to compare the effects of vehicle and compound 52 on responses evoked by mechanical and heat stimuli. All post-hoc comparisons between groups or over time were made using Newman-Kuels tests. All statistical analyses were performed using Sigma Stat software (Systat Software, San Jose, CA). A probability value < 0.05 was considered significant. All data are presented as mean (±S.E.M).

## Results

### A series of aminotriazines decreased formalin-induced flinching behavior

We have previously shown that compound 52 is effective in the formalin model of pain, for which the readout is spontaneous flinching arising in part from sensitization of the spinal cord to noxious chemical peripheral inputs [23]. Compound 52 had minimal activity in extensive profiling against panels of GPCRs, kinases, and the full Cerep panel [18]. Efficacy beyond a single molecule to a series of sodium channel inhibitors would strengthen greatly the case that the analgesic efficacy was indeed via sodium channel inhibition. Accordingly, each of a series of nine aminotriazines with properties suitable for *in vivo* studies, compound 52 and aminotriazine congeners labeled compounds A through H (Fig 1), was characterized *in vitro* and in the formalin test (Table 1). Compounds all were strongly state dependent inhibitors of Nav1.7, with potency on the order of several hundreds of nanomolar to single digit micromolar as tested with holding voltages producing partial (20%) inactivation. Compounds all had much weaker potency, often beyond the testable solubility limit, with holding voltage protocols sufficiently negative that no inactivation was produced. Significant differences among compounds were seen in the amount of CNS penetration: *in vivo* brain to plasma concentration ratios ranged from approximately 50% to 3%, essentially zero as this measure includes blood vessels within the brain.

**Table 1:** *In vitro* and *in vivo* properties of aminotriazines used in this study.

Compound	IC50 (μM) Non-inactivated	IC50 (μM) 20%-inactivated	IC50 (μM) Rat 20%-inactivated	Plasma protein binding (%)	[Brain] / [plasma]	Analgesic efficacy	Effective [plasma]
**52**	**3.6**	**0.17**	**0.39**	**97.3**	**0.3**	**Yes**	**1.93 μM**
**A**	**>10**	**0.19**	**0.52**	**98.7**	**0.1**	**Yes**	**3.02 μM**
**B**	**>10**	**0.5**	**3.5**	**99.4**	**ND**	**Yes**	**8.75 μM**
**C**	**>10**	**1.3**	**4.9**	**83.6**	**0.03**	**Yes**	**3.68 μM**
**D**	**>10**	**0.6**	**1.7**	**93.3**	**0.19**	**Yes**	**1.27 μM**
**E**	**2.1**	**0.09**	**3.5**	**99.1**	**0.49**	**Yes**	**6.14 μM**
**F**	**>10**	**0.18**	**1.0**	**99.9**	**0.33**	**Yes**	**3.70 μM**
**G**	**6.6**	**0.3**	**0.80**	**99.4**	**0.09**	**No**	**-**
**H**	**>10**	**0.66**	**2.2**	**95.5**	**ND**	**No**	**-**

Shown for each compound are IC50 on non-inactivated human Nav1.7, IC50 on 20%-inactivated human Nav1.7, and IC50 on 20%-inactivated rat Nav1.7, all taken with patch-clamp electrophysiology; *in vitro* plasma protein binding; the ratio of brain to plasma concentrations *in vivo*; whether the compound produced analgesic efficacy in the rat formalin model of pain; and the plasma concentration corresponding to the lowest dose that produced efficacy. IC50s on hNav1.7 were measured with manual patch-clamp electrophysiology; IC50s on rNav1.7 were measured with the PatchXpress® automated electrophysiology platform. Brain to plasma ratios were calculated from concentrations experimentally measured following the formalin test. ND = no data. Analgesic efficacy was determined by a statistically significant (p < 0.05) decrease in formalin-induced flinching for which the same dose did not produce a reduction in movement in the open field assay that obviated the formalin result. Effective [plasma] is the mean (n = 8, except n = 7 for compound E) terminal plasma concentration produced by the lowest effective dose of each compound.

**Fig 2:**
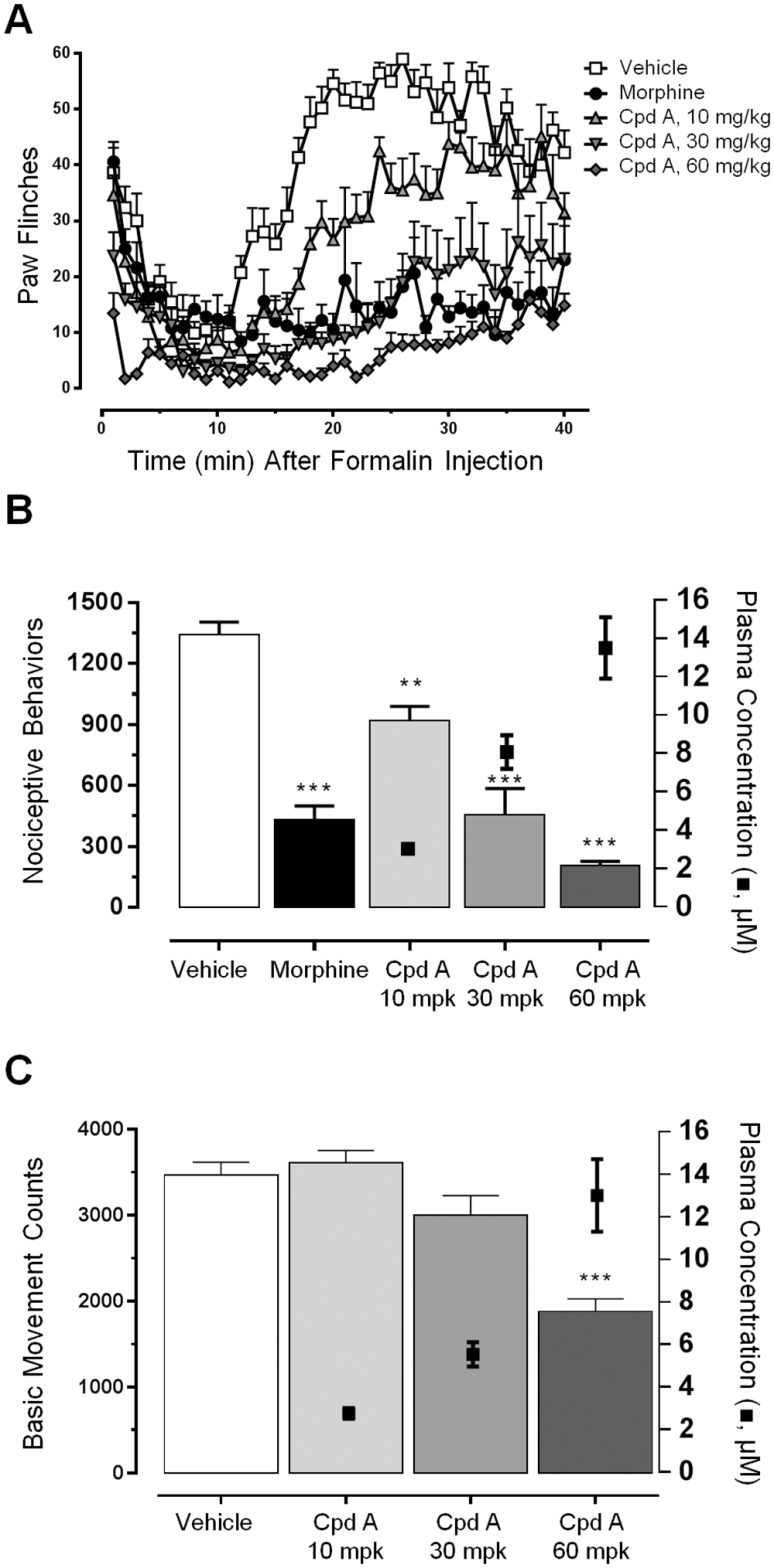
Testing of representative aminotriazine compound A in the formalin model of pain. **A**, Timecourse of flinching binned every minute for each of five cohorts: vehicle, positive control morphine (2 mg/kg), and 10 mg/kg, 30 mg/kg, and 60 mg/kg compound A. All cohorts were n = 8 animals, except morphine was n = 5. **B**, Total flinches in phase 2 (10 minutes to 40 minutes) (bars and left y-axis) and terminal plasma concentrations of compound A (symbols and right y-axis). Total reduction in phase 2 flinching as percent of vehicle: morphine 68%, 10 mg/kg compound A 32%, 30 mg/kg compound A 66%, 60 mg/kg compound A 85%. Terminal plasma and brain concentrations of compound A: for the 10 mg/kg dose plasma 3.02 uM ± 0.21 μM and brain 0.28 μM ± 0.029 μM; for the 30 mg/kg dose plasma 8.07 μM ± 0.65 μM and brain 0.88 μM ± 0.14 μM; for the 60 mg/kg dose plasma 13.5 μM ± 1.60 μM and brain 1.54 μM ± 0.10 μM (for each dose cohort mean ± SEM, plasma n = 8, brain n = 4). **C**, Effects of compound A on spontaneous locomotor behaviors. Graph shows basic movement counts (bars and left y-axis) and terminal plasma concentrations (symbols and right y-axis) following indicated doses of compound A. Total percent reduction and the corresponding terminal plasma concentrations: for the 10 mg/kg dose -4.2% and 2.75 μM ± 0.26 μM; for the 30 mg/kg dose 14% and 5.53 μM ± 0.57 μM; for the 60 mg/kg dose 46% and 13.0 μM ± 1.7 μM (brains were not analyzed) (for each dose cohort mean ± SEM, n = 8). **p < 0.01, ***p < 0.001.

*In vivo* effects of a representative aminotriazine (compound A, different from compound 52) in the formalin model are shown in Fig 2. Total flinching in phase 2 (10 to 40 minutes) decreased with compound in a dose-dependent and plasma concentration-dependent manner (F_4,30_ = 35.2, p < 0.0001)(Fig 2b). All compounds were tested in this manner, and a statistically significant and plasma concentration-dependent reduction in formalin flinching also was seen on tests of compound 52 and compounds B, C, D, E, and F (S1 Table). Compounds G and H did not achieve statistically significant reversals in formalin flinching. These were not outliers in terms of Nav1.7 potency, peak plasma concentration, pharmacokinetics, or *in vitro* protein binding, and absent a direct measure of target occupancy we do not know why they were not effective. In all formalin studies, morphine was used as a positive control and produced a statistically significant decrease in flinching compared to the vehicle group.

To control for nonspecific pharmacological reductions in movement that might confound the formalin pain behavioral readout, all doses of all effective compounds were tested in parallel on naïve rats for effects on spontaneous locomotor behavior. Compound A did not significantly reduce basic movement at 10 mg/kg and 30 mg/kg doses (4.2% increase and 13.5% decrease, compared to the 31.4% and 66.1% reduction in formalin flinching at these doses). It did produce a statistically significant reduction in movement of 46% at the 60 mg/kg dose (F_3,28_ = 21.42, p < 0.0001) (Fig 2c). To determine what reduction of basic movement could interfere with a formalin readout, the sedative diazepam was tested in the formalin and open field tests. Diazepam dosed at 10 mg/kg was the lowest dose that gave a statistically significant (22%) reduction in formalin-induced flinching, and this dose produced a 67% reduction in basic movement. Accordingly, we considered compound A an effective analgesic, as the 10 mg/kg and 30 mg/kg doses produced reductions in flinching and not significant reductions in basic movement, and the 46% reduction in basic movement at the highest dose was not high enough to negate the 84.6% reduction in flinching at this dose. Results for compounds B-F in the open field assay are given in S1 Table. Table 1 summarizes the compounds that were considered effective analgesics by a combination of efficacy in the formalin test and minor or insignificant effects in the open-field basic movement assay. *In vitro* potencies within the series did not vary enough to assess a true PK / PD relation, but efficacy from many congeners of a series of sodium channel inhibitors supports the putative mechanism of analgesic efficacy via sodium channel inhibition. Despite structural similarity and close *in vitro* potencies, this series of aminotriazines did show a range of brain to plasma ratios, ranging from 0.49 (compound E) to 0.03 (compound C, approximately the quantitation limit). Note that compounds with very poor brain penetration nevertheless produced efficacy.

### Compound 52 decreased thermal hyperalgesia produced by inflammation

**Fig 3:**
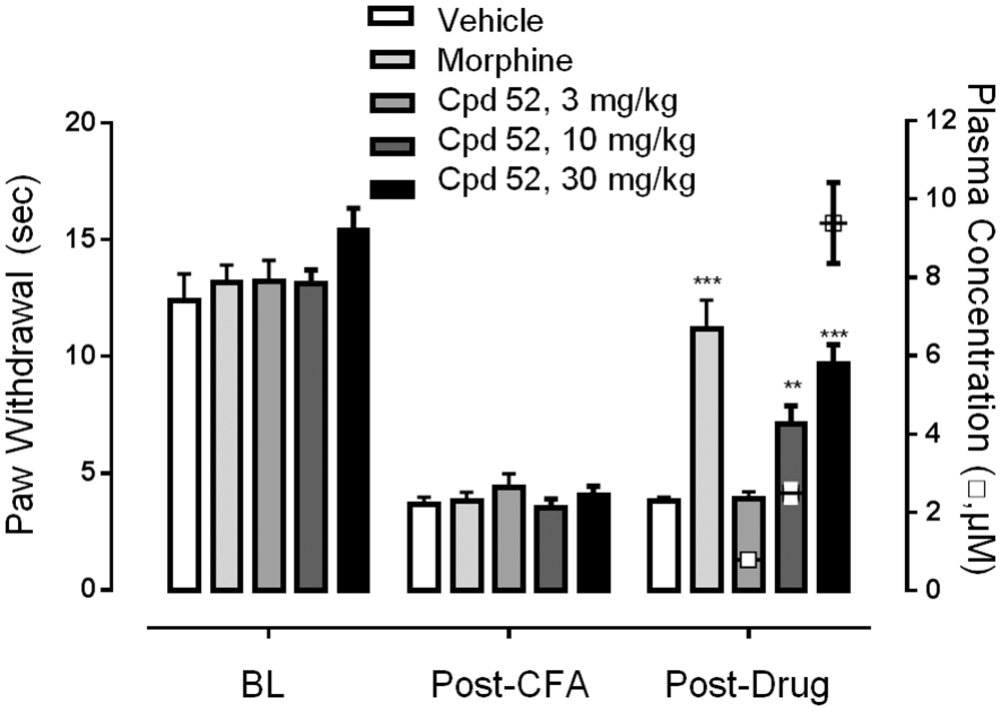
Compound 52 produced dose-dependent efficacy against thermal hyperalgesia in the CFA model. Paw withdrawal latency (bars and left y-axis) and terminal plasma concentration of compound 52 (hollow square symbols and right y-axis) for each cohort of animals is shown at baseline (BL), 24 hours following CFA injection (Post-CFA), and 24 hours following CFA injection after administration of either vehicle, morphine, or the indicated dose of compound 52 (Post-Drug). Data are mean ± SEM, n = 7–10 animals per cohort. Average reversal for each condition compared to baseline: morphine 87%, 10 mg/kg compound 52 40%, and 30 mg/kg compound 52 53%. Terminal plasma and brain concentrations of compound 52: for the 3 mg/kg dose plasma 0.78 μM ± 0.042 μM (n = 8) (brains not analyzed); for the 10 mg/kg dose plasma 2.48 μM ± 0.22 μM (n = 7) and brain 0.81 μM ± 0.063 μM (n = 3); for the 30 mg/kg dose plasma 9.39 μM ± 1.03 μM (n = 7) and brain 2.36 μM ± 0.38 μM (n = 3) (all values mean ± SEM). **p < 0.01, ***p < 0.001.

We profiled compound 52 in an additional behavioral test reflecting a more persistent hyperalgesia, perhaps corresponding more to physiological inflammation-induced pain. Administration of CFA into the paw was followed by robust thermal hyperalgesia 24 hours after injection as indicated by a decrease in paw withdrawal latency (Fig 3, F_1,9_ = 50.43, p < 0.0001). Compound 52 reversed the decrease in withdrawal latency in a dose-dependent and concentration-dependent manner, with maximum effect approaching the morphine positive control (F_4,35_ = 16.44, p < 0.0001). Plasma concentration of 2.48 μM± 0.22 μM (mean ± SEM, n = 7) in the 10 mg/kg dose cohort was the lowest concentration that produced efficacy in the CFA model (compare to Table 1 for corresponding concentration in the formalin test).

### Compound 52 did not significantly decrease tactile allodynia produced by nerve injury

Following spinal nerve ligation (SNL) surgery, animals developed robust tactile allodynia. At 21 days after SNL, average withdrawal thresholds decreased from 12.6 g before nerve ligation to 1.6 g. After drug treatment, the positive control gabapentin gave a 44% reversal of tactile allodynia, and this was statistically significant (F_2,44_ = 101.1, p = 0.0078, n = 9, Bonferroni’s multiple comparison test) compared to the vehicle group. A 30 mg/kg dose of compound 52 gave a 15% reversal of tactile allodynia compared to the vehicle group, but this was not statistically significant (p = 0.99, n = 8, Bonferroni’s multiple comparison test).

### Mechanistic studies: General characteristics of nociceptors

To understand further the mechanism by which aminotriazines produced analgesia, we examined the effects in living rats of compound 52 administered systemically on the response properties of nociceptors sensitized by CFA. Peripheral sensory nerves commonly are classified based on axonal conduction velocity as C-fibers (slowest), Aδ-fibers (intermediate), and A-beta fibers (fastest), with functional subtypes defined by additional categories including the sensory stimulus to which they respond. Extrapolating from the formalin test that effects of aminotriazines seem to be exerted through peripheral neurons, recordings were made from a total of 55 nociceptors whose receptive fields were located on the plantar surface of the inflamed hind paw. Of these, 33 were C-fiber nociceptors and 22 were Aδ-fiber nociceptors. The mean conduction velocity for C-fiber nociceptors was 1.04 ± 0.09 m/s, whereas Aδ-fiber nociceptors had a mean conduction velocity of 6.9 ± 1.6 m/s. Mechanical response thresholds were determined using von Frey monofilaments, and mean thresholds were 48.4 ±4.8 mN for C-fiber nociceptors and 32.9 ± 5.2 mN for Aδ-fiber nociceptors. Seventy-eight percent (26/33) of C-fiber and 73% (16/22) of Aδ-fiber nociceptors exhibited ongoing spontaneous activity. The rate of ongoing activity ranged from 0.03 to 2.3 Hz for C-fiber nociceptors and from 0.2 to 1.2 Hz for Aδ-fiber nociceptors. Since we were interested in the effects of compound 52 on ongoing spontaneous activity and sensitization, only nociceptors that exhibited an ongoing rate of ongoing activity > 0.1 Hz were studied.

### Compound 52 attenuated sensitization of C-fiber nociceptors

**Fig 4:**
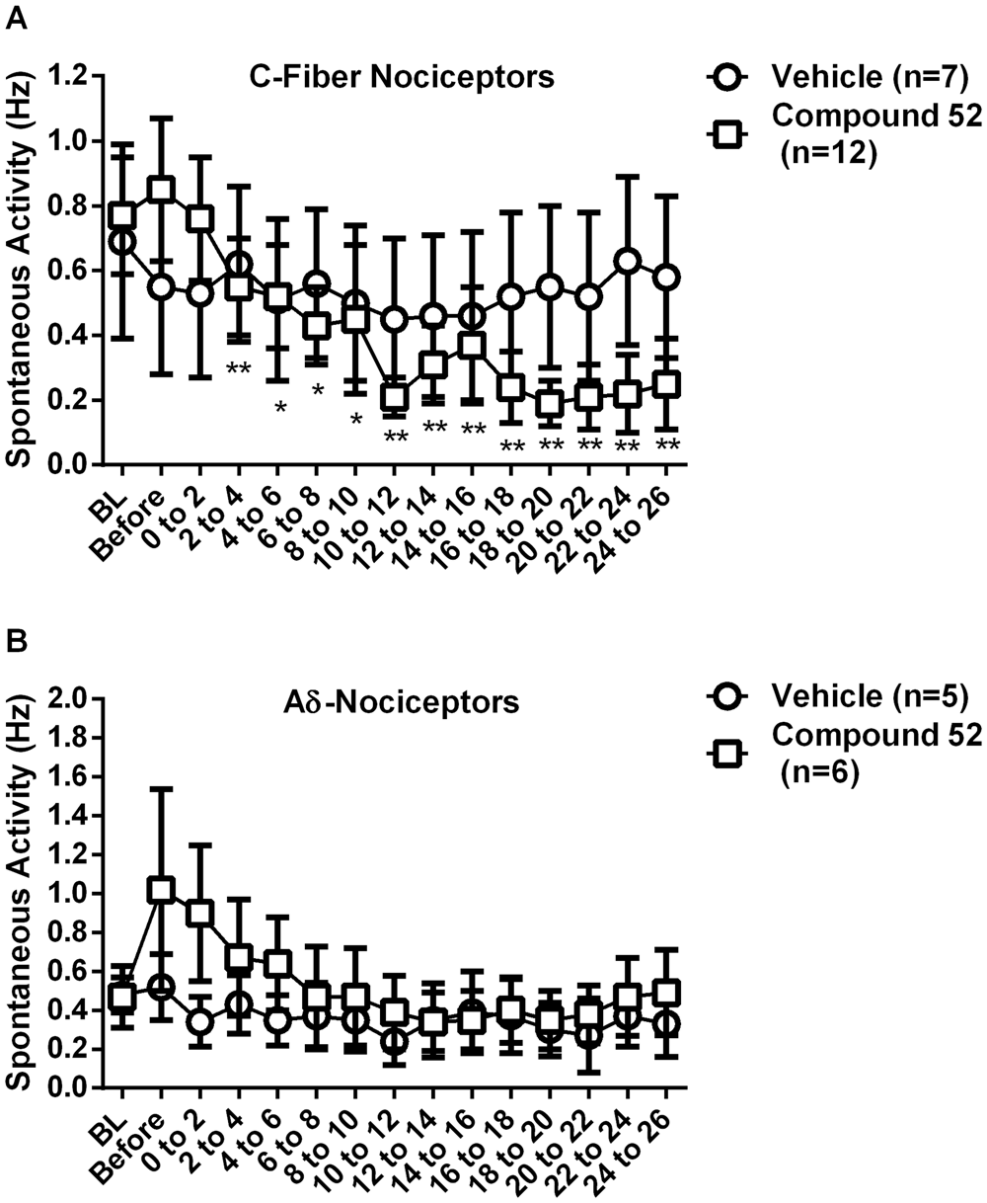
Compound 52 reduced the ongoing spontaneous activity of C-fiber nociceptors but not Aδ-fiber nociceptors. Intravenous injection of compound 52 decreased ongoing spontaneous activity of C-fiber nociceptors sensitized by intraplantar administration of CFA (upper panel). The level of ongoing spontaneous activity of sensitized C-fiber nociceptors was significantly lower following administration of compound 52 than following administration of vehicle, from 12 minutes post-administration on. The attenuation of ongoing spontaneous activity by compound 52 continued until the end of the monitoring period (26 minutes after administration of drug). In contrast, administration of compound 52 did not decrease the level of ongoing spontaneous activity in Aδ-fiber nociceptors when compared to vehicle (lower panel). *p < 0.05; **p < 0.01.

Compound 52 reduced the ongoing spontaneous activity of C-fiber nociceptors. Compound was administered i.v. at a dose that produced maximum analgesia in the behavioral studies. Before injection of vehicle or compound 52, the rate of ongoing spontaneous activity was 0.74 ± 0.17 Hz for C-fiber nociceptors and 0.79 ± 0.29 Hz for Aδ-fiber nociceptors. As shown in Fig 4, the rate of ongoing spontaneous activity in C-fiber nociceptors decreased following injection of compound 52 (n = 12) but not vehicle (n = 7) (repeated measures ANOVA, F_14, 238_ = 4.54, p < 0.01). The rate decreased significantly by 12 minutes after beginning the injection of compound 52 and remained decreased until the end of the recording period at 26 minutes (Newman-Keuls, p < 0.01). In parallel studies, compound 52 was verified to give behavioral analgesic efficacy equivalent to morphine in the formalin model of pain, using the same formulation and i.v. delivery route that was used for electrophysiological studies (data not shown). For Aδ-fiber nociceptors, injection of vehicle or of compound 52 did not reduce the rate of ongoing spontaneous activity (Fig 4).

**Fig 5:**
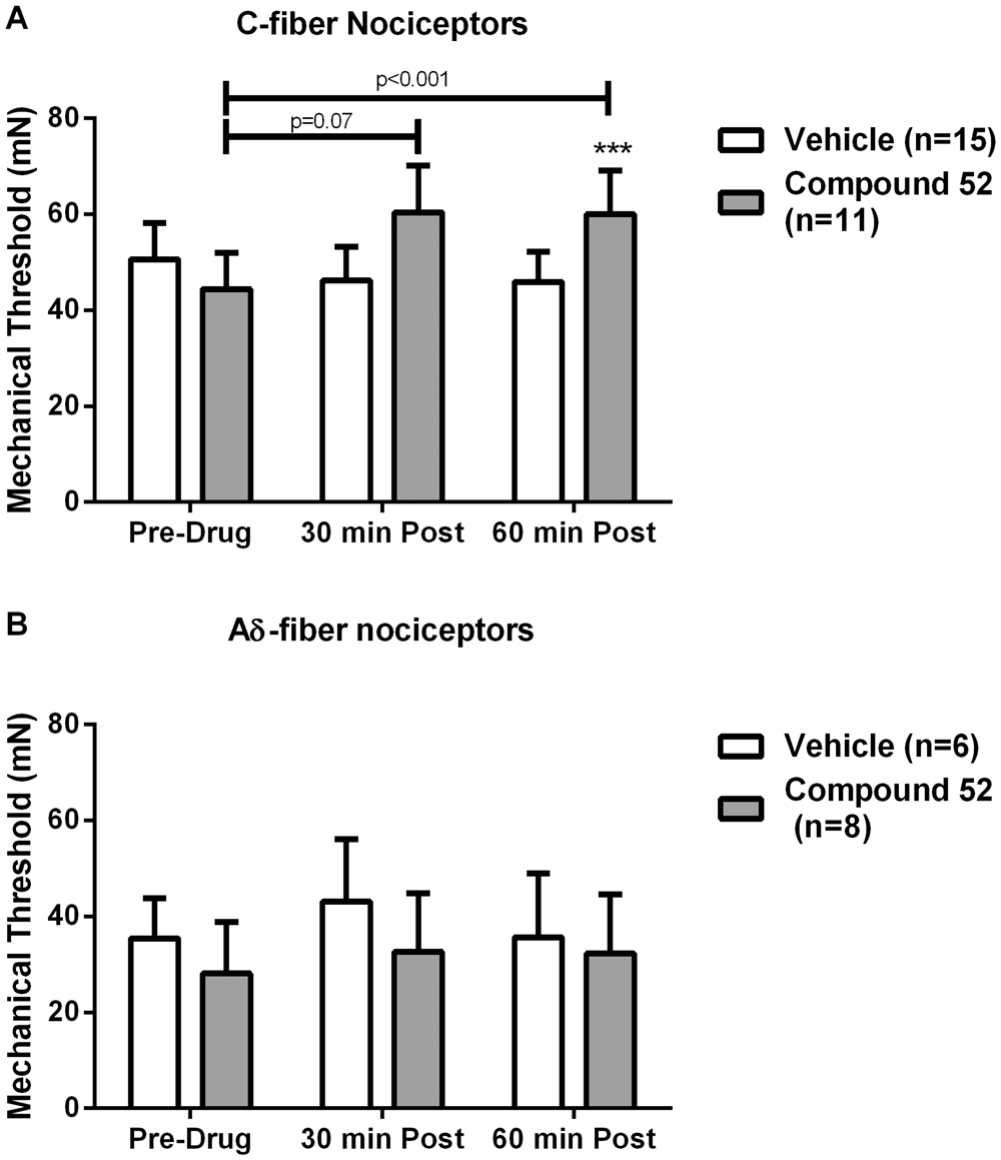
Compound 52 attenuated CFA-induced sensitization of C-fiber nociceptors but not Aδ-fiber nociceptors to mechanical stimuli. **A**, Mechanical response thresholds of sensitized C-fiber nociceptors increased at 30 minutes (p = 0.07) and 60 minutes (p < 0.001) following intravenous injection of compound 52. Administration of vehicle did not alter mechanical response thresholds in sensitized C-fiber nociceptors. **B**, Compound 52 did not attenuate CFA-induced sensitization of Aδ-fiber nociceptors, as mechanical response thresholds were not significantly different from pre-drug levels following administration of compound 52 (lower panel). ***p < 0.001.

Compound 52 also attenuated CFA-induced sensitization of C-fiber nociceptors to mechanical stimuli (Fig 5). Before injection of vehicle (n = 15) or compound 52 (n = 11), mechanical response thresholds of C-fiber nociceptors did not differ between the groups (50.6 ± 7.5 mN before vehicle and 44.5 ± 7.5 mN before compound 52). Injection of vehicle did not alter mechanical thresholds, whereas there was a small increase in mechanical threshold (two-way repeated measures ANOVA F_2,44_ = 4.53, p < 0.05) at 30 (60.4 ± 9.8 mN; Newman-Keuls, p < 0.05) and 60 (60.1 ±9.1 mN; Newman-Kuels, p < 0.05) minutes after compound 52. Neither vehicle nor compound 52 altered mechanical response thresholds of Aδ-fiber nociceptors.

In addition to elevating mechanical response thresholds, compound 52 tended to reduce the number of action potentials evoked by a suprathreshold mechanical stimulus (250 mN), although this was not significantly different from the vehicle-treated group (two-way repeated measures ANOVA, p = 0.15). The mean number of evoked action potentials for C-fibers was 28.9 ± 6.2 before compound 52, and 19.9 ± 5.4 and 11.6 ± 3.2 at 30 and 60 minutes after (p < 0.07; n = 8). For the vehicle-treated group, the number of impulses evoked was 39.2 ± 8.8 before, and 35.7 ± 10.1 and 34.2 at 30 and 60 minutes after injection, respectively (n = 11). Responses of Aδ-fiber nociceptors before and after vehicle (n = 5) and compound 52 (n = 7) exhibited a similar trend.

**Fig 6:**
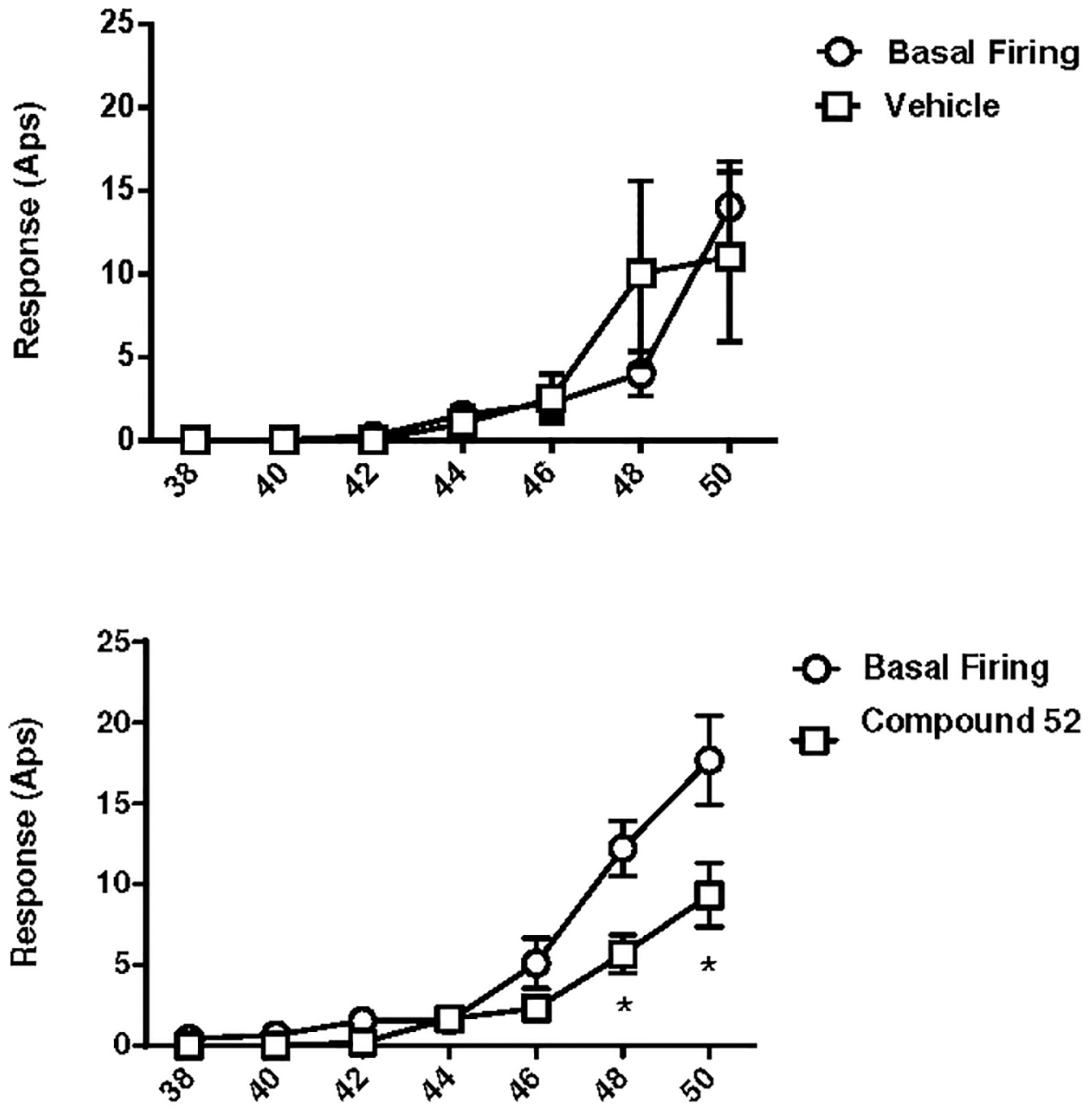
Compound 52 attenuated CFA-induced sensitization of C-fiber nociceptors to heat stimuli. Graphs plot number of action potentials (Aps) in response to thermal stimulus of indicated temperature. Administration of vehicle did not alter heat response thresholds in sensitized C-fiber nociceptors (upper panel). By contrast, heat response thresholds of sensitized C-fiber nociceptors were decreased 30 minutes following intravenous injection of compound 52 (lower panel). The two panels represent separate experiments taken from different cohorts of animals. *p < 0.05.

Responses of C-fiber nociceptors evoked by heat were reduced by compound 52. Heat response thresholds were not changed at 30 minutes following injection of vehicle (43.3°C ± 1.2°C before and 44.4°C ± 0.8°C after; n = 4) or compound 52 (45.0°C ± 1.7°C before and 47.0°C ± 1.3°C after injection; n = 9). However, as shown in Fig 6, compound 52, but not vehicle, reduced the number of action potentials evoked by heat stimuli (two-way repeated measures ANOVA, F_6,132_ = 2.49, p < 0.05). Responses evoked by the most intense heat stimuli, 48°C and 50°C, were reduced at 30 minutes following compound 52 (Newman-Kuels tests, p < 0.05 and p < 0.01, respectively). Also, the cumulative number of action potentials evoked by all heat stimuli was decreased following compound 52, but not vehicle. The mean cumulative numbers of action potentials before and at 30 minutes after injection of vehicle were 22 ± 2.2 and 24.5 ± 10.5, respectively, whereas the cumulative numbers of action potentials decreased from 39.2 ± 6.5 before compound 52 to 19.2 ± 3.6 after (t-test, p < 0.01).

## Discussion

Here we extend our previous report of antinociceptive properties for the Nav1.7 inhibitor compound 52 with more compounds, multiple pain assays, and mechanistic studies of neuronal firing *in vivo*. Comparing the *in vitro* properties of aminotriazines with effects on neuronal and behavioral measures allows inferences as to mechanisms governing pain and to the molecular pharmacology that produces analgesia in these models. Results suggest mechanisms by which Nav1.7 activity encodes noxious stimuli and may set guideposts for the development of future sodium channel-targeted drugs.

The series efficacy presented here strengthens the case that aminotriazines do exert analgesic efficacy via sodium channels, likely Nav1.7, and that the previously reported compound 52 is not an outlier. This hypothesis is not airtight, however, since these compounds do not inhibit just Nav1.7 among all sodium channels; a specific inhibitor of rat Nav1.7 with complete isoform selectivity and with properties suitable for *in vivo* studies has not been described. Compound 52 was significantly more potent on TTX-S sodium channels than on native and cloned TTX-R sodium channels [18], but given the uncertainties on channel gating states in the *in vivo* situation data presented here do not exclude a contribution of TTX-R channels in mediating pain. Rather, we regard Nav1.7 as the most likely target of aminotriazines from genetic evidence showing that Nav1.7 knockout mice are unresponsive in models of pain, including in formalin and CFA assays [14]. By comparison, deletion of peripheral sodium channels Nav1.3, Nav1.8, and Nav1.9 each produces comparatively subtle effects on pain [14, 24–27], and Nav1.3 is not known to be present in adult sensory neurons [28, 29]. A role for Nav1.7 in inflammation-induced pain is consistent with existing literature, in that this channel is increased in sensory ganglia following inflammation [30] and in painful human dental pulp [31], and mice with genetic deletion of Nav1.7 in a subset of neurons exhibited reduced responses to inflammation-induced pain [32]. In sum, data presented here support the hypothesis that pharmacological inhibition as well as genetic deletion of Nav1.7 may alleviate multiple forms of pain [6, 7, 33, 34], and that either the formalin or CFA model in rats is an appropriate driver assay for the development of future Nav1.7 inhibitors.

Sodium channels present multiple conformations amenable to pharmacological modulation, an effect often interpreted via the modulated receptor hypothesis as different potencies for physically different conformations of the receptor [35]. Compound 52 and compound E were effective *in vivo* despite having quite weak *in vitro* potency on non-inactivated Nav1.7, and compounds A, B, C, D, and F were effective *in vivo* despite having *in vitro* potency on non-inactivated Nav1.7 that was too weak to measure (Table 1). Accounting for plasma protein binding, ranging from 83.6% to 99.9% bound, the *in vivo* plasma concentrations giving analgesic efficacy were far too low to have inhibited a significant fraction of non-inactivated sodium channels. Accordingly, we interpret effective compounds as working via inhibition or stabilization of inactivated channels [36]. It is not new that sodium channel inhibitors exert state-dependent inhibition, but preferential inhibition of inactivated states generally is viewed as a means to increase therapeutic window by targeting preferentially sodium channels in pathological tissue rather than as a means to efficacy. Multiple inactivated states for sodium channels exist biophysically and have been shown to govern C-fiber excitability [37–44], and aminotriazines may inhibit or stabilize multiple inactive sodium channel states, including slow or deep states [42, 43]. The efficacy seen with aminotriazines with very low CNS penetration suggests that the relevant channels are on peripheral nerves [45], although we do not have a direct measure of binding to Nav1.7 (or any other receptor) in either the central or peripheral compartment [46]. With selectivity caveats as discussed, we speculate that aminotriazines exert efficacy via Nav1.7 expressed at peripheral nerve terminals, axons, or branch points [37, 47], and that nociceptive signals originating in the periphery are cut off by inhibition of Nav1.7 before reaching the presynaptic terminals in the spinal cord beyond the blood-brain barrier [15, 16, 47]. As the flinch endpoint of the formalin model is thought to reflect increased excitability of the spinal cord [23], this interpretation further suggests that shutting off peripheral pain drivers pharmacologically can reduce some forms of centrally-mediated pain [4]. Nav1.7 also likely can govern release of neurotransmitters in the spinal cord [15, 48] or in other parts of the pain matrix [49]; our experiments do not address whether analgesia could be achieved by inhibition of CNS channels [48].

C-fiber nociceptors have long been known to encode pain and hyperalgesia [50], and our results support existing data showing that C-fiber nociceptors have a strong role in driving the pain of formalin and CFA. Electrophysiological studies *in vivo* have shown that C- and Aδ-fiber nociceptors both are sensitized following CFA [51, 52], and the effects of compound 52 on C-fibers are consistent with previous experiments using a selective peptide inhibitor of Nav1.7 [53] and with effects of a small molecule Nav1.7 inhibitor on ectopic firing of injured sensory nerves [33]. Neither the spontaneous nor the evoked responses of C-fiber nociceptors were completely prevented by compound 52. This is consistent with a preparation taken from the global Nav1.7 knockout in which mechanically-evoked C-fiber firing (not under inflammatory conditions) was reduced but not eliminated [14]. Somewhat surprising was the lack of effect of compound 52 on responses of Aδ-nociceptors, which are also sensitized after CFA [51, 52]. Data suggest that functional Nav1.7 may be primarily associated with C-fiber nociceptors, and that not all C-fiber spiking need be eliminated to produce meaningful analgesia. The small (non-significant) trend towards analgesic efficacy of compound 52 in the nerve injury model parallels the recordings of C-fiber nociceptors, in which compound 52 gave a small (but statistically significant) increase in tactile threshold following CFA sensitization. Whether a small *in vivo* effect actually is present or not, certainly compound 52 has quite subtle effects on the mechanical firing thresholds of C-fiber and Aδ-fiber nociceptors and on behavioral tactile allodynia following SNL, consistent with data showing a higher proportion of Aδ-fibers than C-fibers respond to mechanical stimulation [50]. A role for Nav1.7 in neuropathic or persistent pain is suggested by clinical studies [54–56]; results here suggest that nerve injury and other models that use tactile stimuli to elicit an endpoint of behavioral allodynia may not predict the effects of Nav1.7 blockers on clinical neuropathic pain.

Data presented here suggest that targeting inactive states of Nav1.7 decreases spontaneous firing of peripheral C-fiber nociceptors, and that this reduces persistent as well as chemical-induced pain. Although the closest pharmacological mimic of human genetic congenital indifference to pain arising from stopgain variants in *SCN9A* is unquestionably a non-state dependent, CNS-penetrant, perfectly Nav1.7-selective inhibitor, data here suggest that Nav1.7-dependent nociceptive transmission can be shut off and pain reduced with peripherally-restricted inhibitors. This would be encouraging news for drug development, as a requirement for CNS penetration imposes additional selectivity hurdles and toxicology concerns. Efficacy of compound 52 in the CFA model likewise suggests that inflammation-induced persistent forms of pain are governed by Nav1.7 and could be alleviated by targeting Nav1.7 pharmacologically [45, 54–56].

## Supporting Information

S1 Table Efficacy of aminotriazines in the formalin model of pain and reductions in basic movement in open-field testing. Listed are reductions for each aminotriazine in phase II of formalin-induced flinching and of overall basic movement. Basic movement testing was done in a formalin-naïve cohort entirely separate from the formalin testing. NS = not significant. ND = not determined. The two values listed in parentheses for a decrease in basic movement, (4.2%) for compound A and (1.4%) for compound D, correspond to a measured increase in basic movement.
(DOCX)
