# Supplementary material for: Inhibition of Inactive States of Tetrodotoxin-Sensitive Sodium Channels Reduces Spontaneous Firing of C-Fiber Nociceptors and Produces Analgesia in Formalin and Complete Freund’s Adjuvant Models of Pain
Source: PLoS One. 2015 Sep 17;10(9):e0138140. doi: 10.1371/journal.pone.0138140 (PMC4575030; doi:10.1371/journal.pone.0138140)
Supplement: S1 Table — Listed are reductions for each aminotriazine in phase II of formalin-induced flinching and of overall basic movement. Basic movement testing was done in a formalin-naïve cohort entirely separate from the formalin testing. NS = not significant. ND = not determined. The two values listed in parentheses for a decrease in basic movement, (4.2%) for compound A and (1.4%) for compound D, correspond to a measured increase in basic movement. (DOCX) [file pone.0138140.s001.docx]

Supplementary Table 1. Efficacy of aminotriazines in the formalin model of pain and reductions in basic movement in open-field testing.

| Compound | Dose (mg/kg) | Decrease in formalin flinching | Significance | Decrease in basic movement | Significance |
| --- | --- | --- | --- | --- | --- |
| 52 | 3 | 9% | NS | 3% | NS |
|  | 10 | 22% | p < 0.05 | 11% | NS |
|  | 30 | 79% | p < 0.001 | 54% | p < 0.001 |
| A | 10 | 32% | p < 0.01 | (4.2%) | NS |
|  | 30 | 66% | p < 0.001 | 14% | NS |
|  | 60 | 85% | p < 0.001 | 46% | p < 0.001 |
| B | 10 | 17% | NS | ND | ND |
|  | 30 | 30% | NS | 5.6% | NS |
|  | 60 | 39% | p < 0.05 | 19% | NS |
| C | 3 | 14% | NS | 6.8% | NS |
|  | 10 | 35% | p < 0.01 | 8.5% | NS |
|  | 30 | 53% | p < 0.001 | 23% | p < 0.001 |
| D | 10 | 36% | p < 0.001 | (1.4%) | NS |
|  | 30 | 62% | p < 0.001 | 28% | p < 0.001 |
|  | 60 | 83% | P < 0.001 | 50% | p < 0.001 |
| E | 10 | 18% | NS | 2% | NS |
|  | 30 | 62% | p < 0.001 | 29% | p < 0.01 |
|  | 100 | 83% | p < 0.001 | 29% | p < 0.01 |
| F | 10 | 0% | NS | 11% | NS |
|  | 30 | 35% | p < 0.01 | 33% | p < 0.01 |
|  | 100 | 67% | p < 0.001 | 54% | p < 0.001 |

Listed are reductions for each aminotriazine in phase II of formalin-induced flinching and of overall basic movement. Basic movement testing was done in a formalin-naïve cohort entirely separate from the formalin testing. NS = not significant. ND = not determined. The two values listed in parentheses for a decrease in basic movement, (4.2%) for compound A and (1.4%) for compound D, correspond to a measured increase in basic movement.
